# Dietary addition of microencapsulated turmeric in an amorphous matrix of maltodextrin on quality characteristics of broiler chicken

**DOI:** 10.5455/javar.2022.i587

**Published:** 2022-06-26

**Authors:** Harvey Febrianta, Vitus Dwi Yunianto, Nurwantoro Nurwantoro, Valentinus Priyo Bintoro

**Affiliations:** Faculty of Animal and Agricultural Sciences, Universitas Diponegoro, Semarang, Indonesia

**Keywords:** Broiler chicken, health status, maltodextrin, microencapsulation, turmeric

## Abstract

**Objective:**

This experiment investigated the effect of microencapsulated turmeric by maltodextrin as an amorphous matrix material on the health status of broiler chickens.

**Materials and Methods:**

The broilers used were 144 healthy 1-day-old males. The average body weight was 47.8 ± 1.42 gm. The statistical design was based on a completely randomized design with four treatments and six replications. There were six broiler chickens in each experimental unit. The treatments were TM0 = 0 gm/kg of basal feed, TM1 = 1 gm/kg of basal feed, TM2 = 2 gm/kg of basal feed, and TM3 = 3 gm/kg of basal feed. The growth performance, physical traits, internal organs, microbial population, intestinal morphology, hematological parameters, and antioxidant profile were examined.

**Results:**

The results reported that microencapsulated turmeric by maltodextrin as an amorphous matrix significantly improved the hematological parameters, growth performance, antioxidant profile, LAB, immune organs, and intestinal morphology. The results also show decreasing coliform and pH of the cecum.

**Conclusions:**

Dietary addition of maltodextrin microencapsulated turmeric of 3 gm/kg in basal feed can be used as a natural feed additive to improve the health status of broiler chickens.

## INTRODUCTION

Feed additives are essential in poultry production, affecting the quality of meat products consumed by the entire populace. The role of additives in poultry feed helps to increase feed efficiency and reduce pathogenic intestinal bacteria. The addition of feed additives is expected to improve broiler growth performance. The antibiotic growth promoters decrease the pathogenic bacteria population in the gut and provide a higher absorptive potential for villi [1]. The obstacle to the use of antibiotic growth promoters is the emergence of residual effects in the bird’s tissues. Health risks can arise for consumers of poultry meat. Therefore, phytogenic additives are needed as a potential replacement for antibiotics.

Turmeric is one of the phytogenic additives added to poultry feed (*Curcuma longa* L.). Turmeric extract can be used as a replacement for antibiotic growth promoters [2]. Turmeric represents 3%–5% of the curcuminoids and is a strong phenolic antioxidant [3]. Due to grinding and heating, active compounds in turmeric, such as curcuminoids, are susceptible to loss and degradation. The degradation of this compound will reduce the phytogenic power of turmeric. The method to protect the phytogenic compound in turmeric can be applied with encapsulation.

Encapsulation is a method by which the encapsulant material is added to protect the active compound in turmeric. In this study, the encapsulant used is maltodextrin because it is highly hygroscopic. Therefore, during the drying process, water quickly evaporates. According to Al-Kassie et al. [4], the production of the encapsulant can protect materials from oxidative damage. Thus, the encapsulation of turmeric ensures the high quality of chicken feed. This experiment aimed to investigate the effect of turmeric (*C. longa *L.) microencapsulation by maltodextrin as an amorphous matrix on the hematological indices profile, intestinal morphology, and growth performance of broiler chickens.

## Materials and Methods

### Ethical approval

This experiment was conducted following the regulations in Animal Health of the Indonesian Law on Livestock and Animal Health (UU/18/2009, Article 80).

### Microencapsulated turmeric preparation

The turmeric (*C. longa *L*.*) and maltodextrin originated from Yogyakarta, Indonesia. First, the herbal grinder was used to grind the turmeric to a fineness level of 100 mesh. Then, 100 ml of ethanol solvent was added to extract the turmeric powder and put into the evaporator for 4 hours. The extract was mixed with maltodextrin at a 10% total weight concentration as an encapsulant. The extract with the inclusion of encapsulant was then freeze-dried by using an FD-18MTP Labfreez freeze dryer. The samples were milled, and the result was microencapsulated turmeric powder.

### Chicks, diets, and experimental design

A total of 144 male broilers (MB-202 Platinum Sexing) at 1 day of age were purchased from a commercial hatchery. Then, the broilers were weighed (47.83 ± 1.42 gm) and reared for 35 days. Broilers were raised on floor pens (1 × 1 × 1.25 m) equipped with wood shavings, drinkers, and feeders. The formulation of the basal diet (mash form) is shown in Table 1. The chicks were treated with microencapsulated turmeric in an amorphous matrix of maltodextrin as follows: TM0 = 0 gm/kg of basal feed, TM1 = 1 gm/kg of basal feed, TM2 = 2 gm/kg of basal feed, and TM3 = 3 gm/kg of basal feed.

### Scanning electron microscopy

The turmeric microcapsule was identified using an analytical (SEM-EDX JEOL JSM-6510LA) vacuum mode at 10 kV, and a scale bar at 5 μm.

### Growth performance

The growth performance parameters were based on the method described by Johannah et al. [5], with a few modifications. Briefly, the chicks (1 day of age) were weighed, then the chickens were evaluated weekly based on their feed intake and body weight for each replicate in the groups until day 35. Additionally, 24 chickens were fed with basal feed combined with 0.3% of Cr2O3 for 3 days. Then, the excreta were collected to measure protein intake and digestion [6].

### Physical characteristics and inner body organ analysis

Six birds at 35 days of age, from each treatment representing all replication, were randomly taken and slaughtered to determine physical characteristics (pH, color, tenderness, and WHC) and inner body (proventriculus, liver, heart, pancreas, and intestinal morphology) based on the method described by Attia et al. [7]. Additionally, the lymphoid organs were removed and weighed using a digital scale (Sartorius BL 1500). Then, the results were reported as inner body organ weight (%).

### Microbiological analysis

The microbiological analysis was conducted according to that described by Peng et al. [1], with a few modifications. For lactic acid bacteria analysis, a 1-gram sample of digesta from cecum and ileum was diluted in a sodium chloride physiological solution (9 ml). Then, the mixture was diluted with de Man, Rogosa, and Sharpe agar until it reached 10^-8^, and it was incubated with an incubator (Memmert Incubator, IN55) for 48 h at a temperature of 37°C. For coliform analysis, the mixture sample was diluted until 10^-6^ and plated on lactose broth (LB) agar. Then, it was incubated in an incubator (Memmert Incubator, IN55). The total bacterial population was calculated as log CFU/gm of digesta. Furthermore, 1-gm of digesta sample from the cecum and ileum was diluted with aquadest (10 ml). Then, a pH meter (OHAUS ST300 portable pH meter) was used to measure the pH of the mixture.

**Table 1.** Ingredient and nutrient composition of basal feed.

Ingredient (%)	Starter-grower (1–27 days)	Finisher (28–35 days)
Rice bran	5.54	5.86
Corn	59.50	63.94
Soy bean meal	26.40	22.40
Fish meal	7.56	6.80
Premix^a^	1.00	1.00
Total basal feed	100.00	100.00
**Nutrient composition**		
Metabolizable energy (kcal/kg)	3,084.62	3,176.53
Crude protein (%)	21.51	18.74
Crude lipid (%)	5.53	5.16
Crude fiber (%)	3.89	3.91
Calcium (%)	0.87	0.82
Arginine (%)	1.24	1.21
Lysine (%)	1.12	1.09
Phosphorus (%)	0.48	0.45
Methionine (%)	0.35	0.31

a Contained per kilogram of premix: 230,000 IU Vitamin A; 72,000 IU vitamin D_3_; 7 mg Vitamin B_12_; 5000 mg calcium D pantothenat; 6000 mg Fe; 4000 mg Mn; 3500 mg Zn; and 300 mg Cu.

### Histological sample preparation and intestinal morphometric analysis

Intestinal morphology analyses were according to that described by Prakatur et al. [8], with a few modifications. The analyses were conducted using the villi to crypt depth ratio, crypt depth, and villi height. The 10% neutralized formalin was used to fix the samples of the chickens directly after slaughter. The intestinal samples (duodenum, jejunum, and ileum) were dehydrated with ethyl alcohol (90%), embedded in paraffin, and cleared with xylene. The paraffin block was cut with a microtome at 4 μm thick per chicken intestinal sample. Then, the samples were stained with eosin and hematoxylin. In brief, the samples were examined under a 4x magnification Leica ICC50HD microscope. The software used to measure morphometrics was Leica Application Suite version 3.4.0.

### Hematological and antioxidant analysis

The blood samples of six chickens at 35 days of age were collected from the wing veins, then placed into ethylenediaminetetraacetic acid tubes and centrifuged at 1,500 rpm for 10 min (4°C). The serum was stored in Eppendorf vials at a temperature of −18°C for further analysis. The serum levels were determined based on colorimetric methods by using a spectrophotometer (Shimadzu UV-2600). Additionally, the red blood cell count was determined by using the hemocytometer method, hemoglobin (Hb) was determined using cyanmethemoglobin, and packed cell volume was measured based on microhematocrit methods [7].

### Statistical analysis

All results were calculated using the SAS 9.2 software program, according to a completely randomized design. Four treatments and six replications were used for all parameters.

## Results and Discussion

### The morphology of turmeric microcapsules

**Figure 1.**
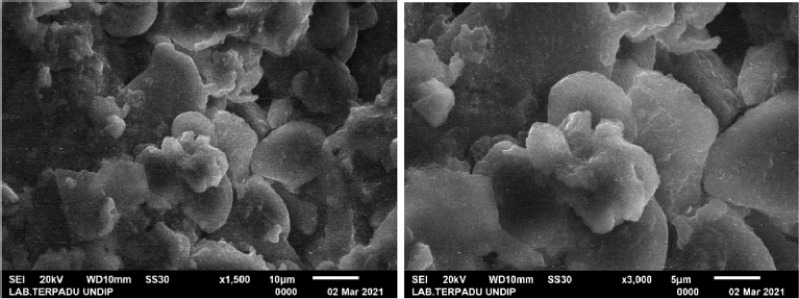
SEM image (1,500×) and (3,000×) of turmeric microencapsulation.

Based on Figure 1, it can be observed that the microcapsule morphology of turmeric was shrink-shaped and round. Some structures were amorphous, most likely due to maltodextrin as a coating material. This is presumably because the process of water evaporation and drying time causes the form of microcapsules to shrink and have a round shape. This result is in line with Jiayue et al. [9], who stated that the rapid evaporation caused the microcapsule structure’s concave shape at the drying time. Also, Liu et al. [10] said that a microcapsule’s quality is not affected by how its structure changes as it shrinks.

### Growth performance

The addition of 3 gm/kg microencapsulated turmeric in basal feed (TM3) resulted in a significant (*p* < 0.05) increase in broiler body weight (BW), feed intake, protein intake, and protein digestibility, followed by TM2, TM1, and TM0, as listed in Table 2. However, the feed conversion ratio (FCR) indicated a lower result (*p* < 0.05) when the addition of microencapsulated turmeric was increased. This is presumably because basal feed with the addition of treated turmeric (TM) increased the appetite of the chickens. Our findings are in accordance with the results of Adegoke et al. [3], who stated that the addition of turmeric (*C. longa L*.) powder (5 gm/kg) in the basal feed improved the growth performance of chicks. Furthermore, Johannah et al. [5] stated that turmeric extract containing curcumin and turmerones improved the body weight gain of broilers. Hofman et al. [11] said that maltodextrin is made by breaking down starch and can give one energy and make one hungrier.

**Table 2.** Effects of maltodextrin microencapsulated turmeric on growth performance.

Treatments	Feed intake (gm/bird)	BW (gm/bird)	FCR	Protein intake (gm/bird)	Protein digestibility (%)
TM0	2265^c^	1374^d^	1.64^a^	24.3^d^	68.6^d^
TM1	2360^b^	1464^c^	1.61^b^	26.5^c^	74.8^c^
TM2	2362^b^	1512^b^	1.56^c^	27.9^b^	78.9^b^
TM3	2383^a^	1576^a^	1.51^d^	28.7^a^	82.4^a^
SEM	63.8	21.2	0.095	0.43	1.77
*p *value	0.006	0.002	0.003	0.006	0.011

SEM = Standard error of the mean; TM0 = 0 gm/kg of basal feed; TM1 = 1 gm/kg of basal feed; TM2 = 2 gm/kg of basal feed; and TM3 = 3 gm/kg of basal feed. ^abc^Means in the same column are different at* p < *0.05.

The value of the TM3-treated sample showed the lowest FCR. Our result is in accordance with Rostami et al. [12] and Setyaningrum et al. [13], who stated the higher effect in feed conversion ratio was affected by an increase in feed intake and BW. The value of FCR was also in line with nutrient absorption in the digestive tract and digestion efficiency. The dietary addition of maltodextrin microencapsulated turmeric could increase the nutrient absorption, decrease the FCR value, and increase the intestinal villi height, as listed in Table 6. Shirani et al. [14] reported that higher nutrient digestibility was correlated with the increased value of villi height.

Experimental treatment of TM3 showed the highest protein intake and protein digestibility, followed by TM2, TM1, and control (TM0). The highest value of TM3 could be due to the effect of curcumin as a natural antioxidant and antibacterial agent that could reduce the coliform population and increase villi height. This could cause a significant effect on protein intake and protein digestibility improvements. Our results are in accordance with those studies which said that turmeric contains 5% curcuminoids and is a strong phenolic antioxidant used to stop bacteria from growing and makes it easier for broilers to digest proteins.

### Physical characteristics and inner body organs

The addition of turmeric microcapsules in the basal feed showed significant data on most of the inner body organs (proventriculus, gizzard, liver, and intestinal morphology), as listed in Table 4. Dietary addition of turmeric microcapsules of 3 gm/kg in basal feed (TM3) showed the lowest proventriculus, gizzard, and liver measurements. However, the intestinal value reported the highest result compared to TM0, TM1, and TM2. The decreased values of proventriculus, gizzard, and liver measurements and the increased value of intestinal morphology indicated the improvement of growth performance. Our findings are in accordance with the results of Attia et al. [7], who stated that the increased length of intestinal villi and the lower value of gizzard and proventriculus are the significant effects of the improvement in growth performance. The physical characteristics were not substantial, as listed in Table 3. The result of turmeric is in agreement with Paul et al. [15], who stated that turmeric did not affect some physical components in the thigh meat of the chicken. Escobedo et al. [16] said that the temperature and other environmental conditions affect the different pH levels, color, tenderness, and WHC in chicken meat.

The results of lymphoid organs were greater (*p *< 0.05) in the TM3-treated sample than in control (TM0). The different concentrations of microencapsulated turmeric in the basal feed affected the weight of the bursa of Fabricius and spleen, but not the thymus. The results reveal that the microencapsulation technique protected the bioactive compound in turmeric with maltodextrin as an encapsulant. This treatment can be used as a safe feed additive for broiler chickens. Our findings are in accordance with Paul et al. [15], who stated that adding turmeric powder affected the spleen’s weight index and bursa of Fabricius. Another study [17] reported that turmeric infusion in broilers had the potential to increase the weight of the bursa of Fabricius and spleen. Ahmed et al. [18] said that the weight of the bursa, thymus, and spleen changed when broilers were fed turmeric (*C. longa* L.) and aloe vera.

### Microbial population

As shown in Table 5, the maltodextrin microencapsulated turmeric in the basal feed had a significant effect on the pH of the cecum, coliform, and LAB in the cecum and ileum of broilers. The values of pH and coliform represented a lower result when the broiler was treated with a higher level of microencapsulated turmeric in the basal feed. Our findings are consistent with the study of Erener et al. [19], who stated that natural antibiotics in green tea leaves and turmeric powder significantly reduce the coliform bacteria in the cecum and ileum. Furthermore, Parveen et al. [20] reported that coliform populations in the cecum and ileum were substantially lower when the chickens received basal feed with turmeric powder at 0.5%. Johannah et al. [5] asserted that turmerones and curcumin act as natural antibiotics in the diet and that the compounds can improve meat carcass characteristics. Turmeric has a yellow pigment called curcumin, which has anti-inflammatory, antioxidant, and antimicrobial properties [21].

**Table 3.** Effects of maltodextrin microencapsulated turmeric on the physical characteristics of broilers.

Treatments	pH	Color, optical density	Tenderness (cm^2^/gm)	WHC (cm^2^/gm)	Dressing (%)	Abdominal fat (%)
TM0	5.80	0.191	9.92	18.02	70.1^a^	0.726
TM1	5.92	0.204	10.08	19.62	69.4^ab^	0.701
TM2	6.00	0.214	10.17	20.31	68.5^b^	0.689
TM3	6.04	0.219	10.23	20.76	68.3^b^	0.663
SEM	0.073	0.085	0.066	0.047	0.814	0.045
*p*-value	0.428	0.391	0.274	0.213	0.005	0.562

SEM = Standard error of the mean; TM0 = 0 gm/kg of basal feed; TM1 = 1 gm/kg of basal feed; TM2 = 2 gm/kg of basal feed; and TM3 = 3 gm/kg of basal feed. ^abc^Means in the same column are different at *p <*0.05.

**Table 4.** Effects of maltodextrin microencapsulated turmeric on the inner body organs of broilers.

Treatments	Proventiculus (%)	Liver (%)	Gizzard (%)	Heart (%)	Pancreas (%)	Intestinal (%)	Bursa fabricius (%)	Thymus (%)	Spleen (%)
TM0	0.587^a^	2.35	1.32^a^	0.51	0.276	8.14^b^	0.132^b^	0.362	0.078^b^
TM1	0.483^ab^	2.31	1.27^ab^	0.472	0.257	8.21^ab^	0.145^b^	0.384	0.080^b^
TM2	0.453^ab^	2.26	1.21^ab^	0.489	0.25	8.45^ab^	0.155^ab^	0.376	0.092^ab^
TM3	0.410^bc^	2.18	1.08^b^	0.492	0.221	8.86^a^	0.149^ab^	0.386	0.118^a^
SEM	0.058	0.103	0.056	0.074	0.016	0.051	0.033	0.057	0.038
*p-*value	0.007	0.441	0.003	0.348	0.119	0.01	0.021	0.236	0.006

SEM = Standard error of the mean; TM0 = 0 gm/kg of basal feed; TM1 = 1 gm/kg of basal feed; TM2 = 2 gm/kg of basal feed; and TM3 = 3 gm/kg of basal feed. ^abc^Means in the same column are different at* p <*0.05.

The value of LAB indicated a higher result. A basal feed with the addition of microencapsulated turmeric in an amorphous matrix of maltodextrin can potentially improve the microflora balance in the gastrointestinal tract. The chicken treated with microencapsulated turmeric in basal feed improved intestinal health. The natural compounds curcuminoids, bisdemethoxy curcumin, and demethoxy curcumin in turmeric helped improve the morphophysiology of the intestines in broilers, according to a study [8].

Using maltodextrin as a turmeric coating material demonstrated a potential effect in covering turmeric’s bioactive compounds. Therefore, the encapsulation of turmeric ensured high-quality broiler feed. According to Attia et al. [7], feed additive composition and encapsulant production can protect bioactive compounds from oxidative damage and improve their overall quality. Furthermore, curcumin, an antibacterial compound in turmeric, significantly reduces pathogenic bacteria and enhances protein absorption. Abbas et al. [22] said that the width and height of the villi in the intestine is the main thing that makes nutrients easier to digest.

### Intestinal morphology

The intestinal morphology showed a significantly higher result (*p *< 0.05) in VH and VH : CD, but was not significant in CD, as listed in Table 6. Intestinal morphology parameters showed that maltodextrin microencapsulated turmeric of 3 gm/kg in basal feed (TM3) produced a higher result than TM2, TM1, and control (TM0). A basal feed with the addition of maltodextrin microencapsulated turmeric has the potential to improve microflora balance in the intestinal tract. Dhama et al. [23] stated that curcumin contained in turmeric is known for its antimicrobial action.

**Figure 2.**
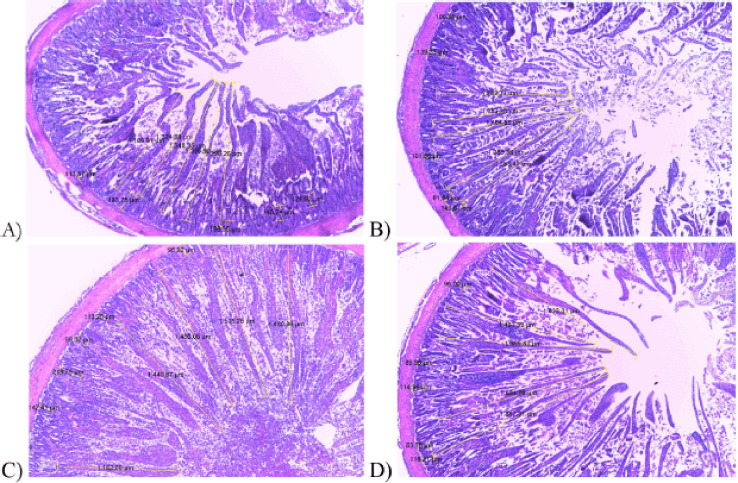
Intestinal morphometric (crypt depth and villi height) of ileum (A), jejunum (B and C), and duodenum (D) with 4× magnification.

The higher level of VH showed that the number of coliform bacteria in the cecum and ileum was decreasing and that the number of lactic acid bacteria (LAB) was rising. This finding is consistent with Shirani et al. [14], who found increased LAB levels in broilers followed high mucin production. Mucin is a glycosylated (glycoconjugates) protein whose main job is to keep pathogenic bacteria from breaking down epithelial cells in the ileum and cecum. The ability to improve the ability to absorb nutrients in the intestine indicated longer intestinal villi than usual. The morphometric measurements are shown in Figure 2. Saeid et al. [24] reported that the longer villi are connected with active mitosis cells, which improves the potential for the absorption of various nutrients. The deeper villi crypts exhibited rapid tissue metabolism to allow the renewal of the intestinal villi [25]. Less crypt depth in the intestinal villi can make it harder for the body to absorb nutrients [26].

**Table 5.** Effects of maltodextrin microencapsulated turmeric on the microbial population of broilers.

Treatments	pH, LAB and Coliform					
	Ileum	Cecum	Ileum	Cecum	Ileum	Cecum
TM0	6.70	6.62^a^	9.04^c^	9.38^c^	8.29^a^	8.20^a^
TM1	6.66	6.56^ab^	9.75^b^	10.15^b^	7.38^b^	7.19^b^
TM2	6.42	6.48^ab^	10.23^ab^	10.50^a^	5.86^c^	6.88^bc^
TM3	6.48	6.37^b^	10.47^a^	10.54^a^	5.84^c^	6.32^c^
SEM	0.102	0.067	0.452	0.364	0.437	0.348
*p-*value	0.388	0.012	0.006	0.004	0.008	0.021

SEM = Standard error of the mean; TM0 = 0 gm/kg of basal feed; TM1 = 1 gm/kg of basal feed; TM2 = 2 gm/kg of basal feed; and TM3 = 3 gm/kg of basal feed. ^abc^Means in the same column are different at *p* < 0.05.

**Table 6.** Effects of maltodextrin microencapsulated turmeric on intestinal morphology.

Treatments	Duodenum, Jejunum and Ileum								
	VH (μm)	CD (μm)	VH: CD	VH (μm)	CD (μm)	VH: CD	VH (μm)	CD (μm)	VH: CD
TM0	1,286^c^	278	4.62^c^	1,272^c^	269	4.37^b^	1,003^c^	228	4.37^c^
TM1	1,569^b^	319	4.92^b^	1,578^b^	308	5.12^a^	1,198^bc^	241	4.97^b^
TM2	1,626^ab^	329	4.94^ab^	1,619^ab^	328	4.92^ab^	1,291^b^	255	5.05^ab^
TM3	1,659^a^	330	5.02^a^	1,647^a^	329	5.01^ab^	1,319^a^	257	5.12^a^
SEM	87.6	31.4	0.27	95.3	28.7	0.25	79.8	21.4	0.22
*p-*value	0.002	0.284	0.001	0.004	0.226	0.001	0.005	0.363	0.006

SEM = Standard error of the mean; VH = Vili height; CD = Crypt depth; VH : CD = Vili height to the crypt depth ratio; TM0 = 0 gm/kg of basal feed; TM1 = 1 gm/kg of basal feed; TM2 = 2 gm/kg of basal feed; and TM3 = 3 gm/kg of basal feed. ^abc^Means in the same column are different at *p* < 0.05.

### Hematological and antioxidant

The values of hematological and antioxidant responses of broilers treated with maltodextrin microencapsulated turmeric are shown in Table 7. Different treatments significantly (*p *< 0.05) affected Hgb and PCV. The increase in Hgb and PCV indicated improved health status in broiler chickens. This result correlates with curcumin being an antioxidant compound in turmeric that was able to increase the iron absorption in the digestive tract of broiler chickens. Sadeghi et al. [17] stated that RBCs and PCV in the broiler increased due to the broiler’s turmeric infusions. Ahmed et al. [18] said that turmeric (*C. longa* L.), which is a phytobiotic, made the health of broilers better.

The addition of maltodextrin microencapsulated turmeric to basal diets had a significant effect on total antioxidant capacity in chickens. Attia et al. [7] stated that the ALKP, ALT, and AST of broilers were reduced by turmeric powder. Broilers’ hematological values were affected by species, sex, age, feed composition, physiological state, and environment temperature [27].

The different turmeric microcapsule treatments reported a significant effect (*p *< 0.05) on the total antioxidant capacity (TAC) and malondialdehyde (MDA). The highest result of TAC was in TM3, followed by TM2, TM1, and TM0. Increased levels of TAC indicated a reduction in hepatocellular leakage, which could positively affect broilers’ health status. The lower result followed the higher TAC in MAD, which showed the reduction of oxidative stress in broilers. Gholami-Ahangaran et al. [28] and Delles et al. [29] reported that turmeric reduced the oxidative stress in broilers fed on diets containing aflatoxin. These antioxidant parameters indicated the improvement of health status when broilers were fed with maltodextrin microencapsulated turmeric of 3 gm/kg of basal feed. Prakatur et al. [8] and Delles et al. [30] reported that antioxidant phenolics increased nutrient absorption.

**Table 7.** Effects of maltodextrin microencapsulated turmeric on the hematological and antioxidant values of broiler chickens.

Treatments	Hematology and antioxidant							
	RBCs (10^6^/mm^3^)	Hgb (gm/dl)	PCV (%)	Alkaline phosphatase (U/l)	ALT (U/l)	AST (U/)	TAC (mmol/l)	MDA (μmol/l)
TM0	1.60	9.7^b^	30.1^b^	9.75^b^	63.4^a^	54.2^a^	408^c^	11.0
TM1	1.58	9.9^ab^	30.7^ab^	10.44^ab^	62.8^a^	53.7^a^	421^b^	10.9
TM2	1.66	10.0^ab^	31.1^a^	11.27^a^	61.5^ab^	53.1^a^	430^ab^	10.8
TM3	1.72	10.06^a^	31.2^a^	11.68^a^	60.4^b^	52.4^ab^	432^a^	10.3
SEM	0.048	0.176	0.253	0.471	0.569	0.388	1.04	0.415
*p*-value	0.098	0.002	0.005	0.010	0.006	0.001	0.001	0.438

SEM = Standard error of the mean; RBCs = Red blood cells; Hgb = Hemoglobin; PCV = Packed cell volume; AST = Aspartate amino transferase; ALT = Alanine amino transferase; TAC = Total antioxidant capacity; MDA = Malnodialdehyde; TM0 = 0 gm/kg of basal feed; TM1 = 1 gm/kg of basal feed; TM2 = 2 gm/kg of basal feed; and TM3 = 3 gm/kg of basal feed. ^abc^Means in the same column are different at* p < *0.05.

## Conclusion

The dietary addition of maltodextrin microencapsulated turmeric of 3 gm/kg in basal feed can be used as a natural feed additive to improve the health status of broiler chickens.

## List of Abbreviations

CFU, Colony forming unit; TM0, Microencapsulated turmeric of 0 gm/kg in basal feed; TM1, Microencapsulated turmeric of 1 gm/kg in basal feed; TM2, Microencapsulated turmeric of 2 gm/kg in basal feed; TM3, Microencapsulated turmeric of 3 gm/kg in basal feed; BW, Body weight, WHC, Water holding capacity; LB agar, Lactose broth agar; LAB, Lactic acid bacteria, EDTA, Ethylenediaminetetraacetic acid; TAC, total antioxidant capacity; SEM, Scanning electron microscope.
